# Pediatric Fractures Are Challenging from Head to Toe

**DOI:** 10.3390/children9050678

**Published:** 2022-05-06

**Authors:** Christiaan J. A. van Bergen

**Affiliations:** Department of Orthopedic Surgery, Amphia Hospital, P.O. Box 90150, 4800 RK Breda, The Netherlands; cvanbergen@amphia.nl; Tel.: +31-76-5955000

Fractures are extremely common in children. The fracture risk is 40% in boys and 28% in girls. Although many pediatric fractures are frequently regarded as “innocent” or “forgiving”, typical complications do occur in this precious population, e.g., premature physeal closure and post-traumatic deformity, which could potentially cause life-long disability.

Despite the high incidence of pediatric injuries, there is still much debate on optimal treatment regimes. Although nonoperative and surgical treatment techniques have developed enormously during the past decades, current management is still more eminence-based rather than evidence-based because of the limited scientific evidence. For example, the recently developed comprehensive Dutch clinical practice guideline on diagnosis and treatment of the most common pediatric fractures included almost solely “low” or “very low” level recommendations, based on the Grading of Recommendations Assessment, Development, and Evaluation (GRADE) criteria. The only exceptions were some forearm fracture recommendations, which received “moderate” GRADEs. There is a clear lack of data and a need for higher-level science in pediatric trauma.

The main goal of this Special Issue in *Children* was to help fill the gap of undiscovered knowledge and improve the scientific understanding of pediatric fractures and related subjects. A great variety of topics were covered in the 14 high-quality original and review papers that have been published so far.

Two studies dealt with general aspects related to acute pediatric trauma. To start with a contemporary hot topic, Verdoni et al. [1] studied the effect of COVID-19 on pediatric emergency department admissions in Italy. Compared to 2019, they found a striking 87% reduction of admissions during the initial COVID period in their observational cohort study. The proportion of children diagnosed with a fracture was significantly higher during the pandemic. In addition, a trend was observed towards more severe injuries and more home-related injuries.

To analyze predictors of mortality in pediatric trauma, Yang et al. [2] retrospectively studied a large population of 1265 children in a trauma center in Taiwan over a 10-year period. The (pediatric age-adjusted) shock index was used in an attempt to predict intensive care admission, surgery and mortality. Interestingly, they found that the index was able to predict the mortality and injury severity in their pediatric trauma population.

In addition, numerous articles investigated specific pediatric fractures, including their diagnosis, treatment, complications and outcomes. In line with the epidemiology of fracture localizations in children, most papers reported on the upper extremity. Many interesting papers were published, from head to toe:

Van der Water et al. [3] provided a comprehensive review on pediatric clavicle fractures and congenital pseudarthrosis. Their article provides useful tools to diagnose, differentiate, and treat both entities.

Plate fixation for proximal humerus fractures was investigated in a small case series by Freislederer et al. [4] from Switzerland. Although closed reduction and K-wire fixation remains the preferred technique for this condition, their series shows that open reduction and plate fixation is a good alternative in cases where closed reduction is unsuccessful.

Supracondylar humerus fractures are very common in children and are sometimes accompanied by brachial artery injuries. Vu et al. [5] described an impressive series of 50 pediatric patients with this unfortunate combination of injuries. They showed that doppler sonography could not reliably identify the vascular lesions, most cases only had vasospasms, and the treatment remained difficult.

In order to investigate when to operate these injuries, Terpstra et al. [6] reviewed the literature on the question of whether there is a benefit of after-hour surgery for supracondylar humeral fractures. Although the included studies showed no differences in functional outcomes, the authors could carefully conclude that surgery during office hours has some advantages with regards to the quality of reduction and fixation.

Next, Hermans et al. [7] investigated the possible association between child abuse and isolated ulnar shaft fractures, as these fractures can result from a direct impact to the forearm when protecting the head. In this retrospective case series of 36 patients from the Netherlands, none of the children were referred to a child protective team. Therefore, a possible association could not be shown.

Forearm fractures may malunite, leading to rotational deficits. Schröder et al. [8] described an interesting case of a patient-specific 3D-guided osteotomy for a malunited forearm, which effectively restored the full range of motion.

With the aim of reducing exposure to radiographic radiation, Zhang et al. [9] in their innovative Canadian study investigated the use of 3D ultrasound and artificial intelligence to diagnose pediatric wrist fractures. The results were remarkable, with very high sensitivity and specificity of the ultrasound scans and a perfect agreement between human judgement and artificial intelligence, suggesting that this technique can reliably rule out these fractures in the emergency room.

One lower limb study was included in this Special Issue. Quality of life after surgery for recurrent patellar dislocation was prospectively studied by Herdea et al. [10] from Romania. A total of 108 pediatric patients were treated by two different soft-tissue surgeries and assessed with the Pediatric International Knee Documentation Committee form. Medial patellofemoral ligament reconstruction led to better quality of life compared to medial imbrication.

After a fracture, the healing of bone in the skeletally immature may be influenced by several factors. Bone healing and the use of non-steroidal anti-inflammatory drugs were systematically reviewed by Choo and Nuelle from the USA [11]. Their analysis of the available literature suggests that the use of these drugs does not increase the risk of nonunion of long bones, although further work is needed with respect to the drug types, dosage and duration.

In another systematic review, Armstrong et al. [12] investigated the orthopedic effects of electronic cigarettes, as they observed delayed unions in their adolescent population using electronic cigarettes. They found no human studies and some experimental studies investigating this topic. Currently, the relationship between electronic cigarettes and bone fractures and healing remains poorly understood, which could be inspiration for further study.

Finally, two papers focused on children with brittle bones. Nijhuis et al. [13] provided a broad perspective on pediatric patients with osteogenesis imperfecta. Among other things, they presented some clear guidelines on the treatment of fractures in this vulnerable population, and underlined the importance of a multidisciplinary approach in dedicated expertise centers.

Ramírez-Vela et al. [14] from Mexico presented an innovative finite element model to study a femur affected by osteogenesis imperfecta. They showed that the highest levels of stress occur in comminuted fractures and the lowest levels in transverse fractures.

In conclusion, this Special Issue contains a great diversity of studies on a broad pediatric population in relation to fractures. Each paper contributes to our knowledge in its own way and helps improve care for our most valuable population. During the writing of this Editorial, more papers are on their way to further fill the current knowledge gaps and identify room for further study.
